# KRAS Mutant Pancreatic Cancer: No Lone Path to an Effective Treatment

**DOI:** 10.3390/cancers8040045

**Published:** 2016-04-18

**Authors:** Daniel Zeitouni, Yuliya Pylayeva-Gupta, Channing J. Der, Kirsten L. Bryant

**Affiliations:** Lineberger Comprehensive Cancer Center, University of North Carolina at Chapel Hill, Chapel Hill, NC 27599, USA; dzeitoun@live.unc.edu (D.Z.); yuliyap1@email.unc.edu (Y.P-G.); bryantkl@email.unc.edu (K.L.B.)

**Keywords:** RAS, pancreatic, cancer, therapeutics

## Abstract

Pancreatic ductal adenocarcinoma (PDAC) is among the deadliest cancers with a dismal 7% 5-year survival rate and is projected to become the second leading cause of cancer-related deaths by 2020. *KRAS* is mutated in 95% of PDACs and is a well-validated driver of PDAC growth and maintenance. However, despite comprehensive efforts, an effective anti-RAS drug has yet to reach the clinic. Different paths to inhibiting RAS signaling are currently under investigation in the hope of finding a successful treatment. Recently, direct RAS binding molecules have been discovered, challenging the perception that RAS is an “undruggable” protein. Other strategies currently being pursued take an indirect approach, targeting proteins that facilitate RAS membrane association or downstream effector signaling. Unbiased genetic screens have identified synthetic lethal interactors of mutant RAS. Most recently, metabolic targets in pathways related to glycolytic signaling, glutamine utilization, autophagy, and macropinocytosis are also being explored. Harnessing the patient’s immune system to fight their cancer is an additional exciting route that is being considered. The “best” path to inhibiting KRAS has yet to be determined, with each having promise as well as potential pitfalls. We will summarize the state-of-the-art for each direction, focusing on efforts directed toward the development of therapeutics for pancreatic cancer patients with mutated *KRAS*.

## 1. Mutant *KRAS* Drives PDAC Development and Maintenance

Approximately 90% of pancreatic cancers are pancreatic ductal adenocarcinoma (PDAC), which is almost universally fatal [1]. Major advancement in the treatment of PDAC has been lacking [2]. Currently, surgery remains the lone curative option. To be eligible for surgery with curable intent the tumor must be resectable, meaning there are no signs of distant metastasis [3]; however, most patients are diagnosed with late-stage disease, and hence less than 20% of patients are eligible. Recent exome sequencing has provided a detailed genetic profile of PDAC, with mutational activation of the *KRAS* oncogene found in ~95% of patients [4,5,6,7]. With significant and compelling evidence that aberrant KRAS protein function is critical for PDAC growth and maintenance [8,9,10], the Pancreatic Cancer Working Group (NCI) identified targeting KRAS as one of four key priorities for pancreatic cancer research [11]. However, despite more than three decades of intensive effort, an effective anti-RAS therapy has yet to reach the clinic [12,13,14].

**Figure 1:**
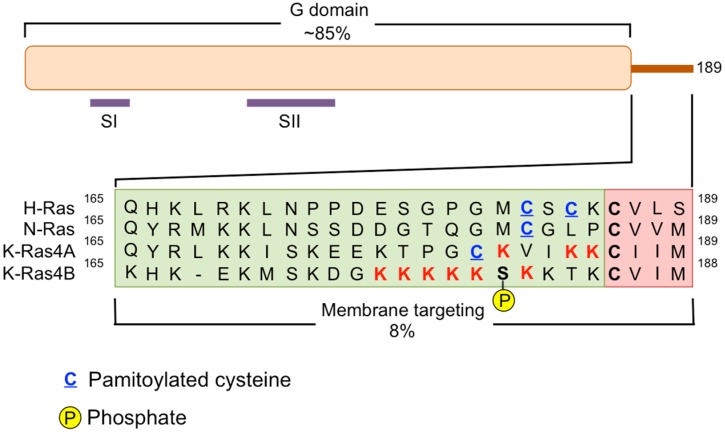
Human RAS proteins are composed two functional domains, the G domain and the membrane targeting domain. The G domain spans residues 1–164 and includes the regions of the protein responsible for binding and hydrolyzing GTP. Specifically, residues in the switch I (SI = amino acids 30–38) region and switch II (SII = amino acids 60–76) region experience a conformational change during GDP-GTP cycling. The membrane targeting domain is comprised of the remaining 24/25 C-terminal residues. The first 20–21 amino acids are referred to as the hypervariable region and this is where the three RAS isoforms exhibit the greatest diversity in protein sequence. The hypervariable region contains elements important for membrane association including cysteines (blue, underlined) that are covalently modified by the addition of a palmitate fatty acid, and stretches of polybasic amino acids. Additionally KRAS4B contains a serine (181) that can be phosphorylated and regulates the association of this protein with the plasma membrane or endomembranes. The four most C-terminal residues of the membrane-targeting domain comprise the CAAX motif, where C = cysteine, A = any aliphatic residue, and X = the terminal amino acid. A C15 farnesyl group is covalently attached to the cysteine residue by farnesyltransferases and this lipid moiety aids in membrane association.

The RAS family of small GTPases includes three genes: *HRAS*, *NRAS*, and *KRAS*. These three loci encode four different protein isoforms: HRAS, NRAS, KRAS4A, and KRAS4B. The two KRAS isoforms differ due to the alternative splicing of exon 4 in the *KRAS* locus, with KRAS4B being the predominant isoform expressed in most tissues [15]. Each RAS protein is comprised of two major domains, the G domain and the membrane targeting domain (Figure 1). All of the isoforms are similar in the amino acid sequence of the G domain (~80%) with major differences being restricted to the hypervariable region of their C-terminal domains [16]. Mutations in RAS occur in residues 12, 13 and 61, and inhibit GTP hydrolysis activity [17]. The three *RAS* genes constitute the most frequently mutated oncogene family in human cancers [14,18]; however, the specific isoform and amino acid mutation varies among cancers. Mutations in *HRAS* are most frequently found in melanoma, bladder and mammary carcinoma; *NRAS* mutations are found in melanoma and thyroid carcinoma; and *KRAS* mutations are most prevalent in cancers of the bladder, ovary, thyroid, lung, colon and pancreas. In pancreatic cancer, mutations in codon 12 of *KRAS* occur the most frequently.

**Figure 2:**
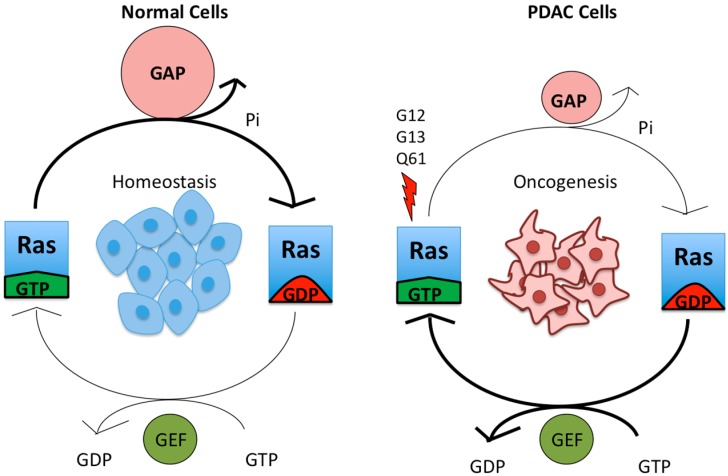
Mutant KRAS is continuously in a GTP-bound, active state. Wild-type KRAS cycles between an active, GTP-bound and an inactive, GDP-bound state, and it exists largely in an inactive state in non-dividing cells. Upon growth factor stimulation, normal KRAS is activated by RAS guanine nucleotide exchange factors (RASGEFs), which facilitate the binding of GTP to KRAS. KRAS-GTP then binds downstream effectors. This signaling is attenuated due to the action of RAS GTPase-activating proteins (RASGAPs), which promote the hydrolysis of the bound GTP to GDP and hence formation of inactive KRAS-GDP. Mutation of residues G12, G13 or Q61 constitutively activates KRAS by preventing the formation of van der Waals interactions between RAS and RASGAPs [20] and interfering with the position of a water molecule necessary for GTP hydrolysis [21], respectively. The arrow thickness and relative size of the symbols for GEFs and GAPs indicate the level of signaling.

RAS proteins play an active role in cell differentiation, proliferation, migration, and apoptosis, making them important in cancer signaling [19]. Individual RAS proteins are activated when they are bound to guanosine triphosphate (GTP) and are inactive when they are bound to guanosine diphosphate (GDP) (Figure 2). Intrinsic RAS GTP-GDP cycling is regulated by guanine nucleotide exchange factors (GEFs) that stimulate nucleotide exchange and by GTPase activating proteins (GAPs) that accelerate the intrinsic GTP hydrolysis activity of RAS (Figure 2). Once activated, RAS-GTP preferentially interacts with a spectrum of catalytically diverse downstream effectors that then regulate a myriad of cytoplasmic signaling networks.

*KRAS* mutation is the initiating genetic event for PDAC, with *KRAS* mutations found in ~95% of pancreatic intraepithelial neoplasias (PanINs), the earliest pre-neoplastic stages of pancreatic cancer progression [22,23]. Progression to invasive, malignant PDAC involves a step-wise accumulation of additional genetic alterations, in particular, the inactivation of tumor suppressor genes [24]. Loss of the cyclin-dependent kinase inhibitor 2A (*CDKN2A*) tumor suppressor gene function by mutation or promoter methylation is found in 95% of pancreatic tumors [25]. *CDKN2A* encodes p16/Ink4a and p14/Arf, inhibitors of cyclin-dependent kinases 4 and 6 (CDK4/6) and MDM2-mediated p53 tumor suppressor degradation, respectively. CDK4/6 hyperactivation in turn inactivates the RB tumor suppressor, promoting tumor progression. Later stage steps involve missense, loss-of-function mutations in *TP53* and the *SMAD4* tumor suppressor genes. *TP53* is mutated in 75% of PDAC. Smad4 functions as a downstream component of the tumor growth factor β-signaling network. In pancreatic cancer, a mutation in Smad4 is often associated with metastatic disease [26].

**Figure 3:**
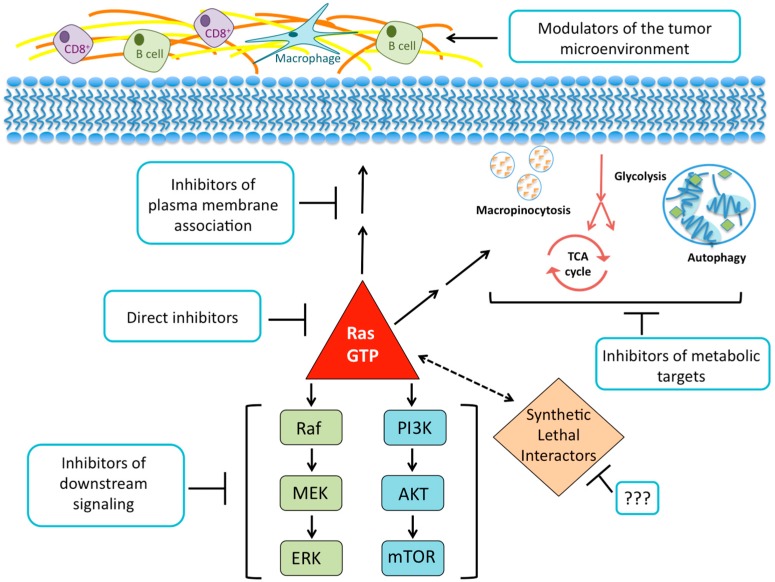
The current paths in the pursuit of an anti-KRAS therapy. There have been past and ongoing efforts to synthesize molecules that bind directly to the RAS protein and inhibit its GDP-GTP regulation or effector signaling. Disrupting RAS membrane localization by inhibiting farnesylation showed promising preclinical effects but no anti-tumor activity in clinical trials. Attempts to inhibit downstream effector signaling have generated a large number of inhibitors currently under clinical evaluation. Unbiased genetic functional RNAi screens have identified genes that may act as synthetic lethal interactors. However, these studies have been limited by reproducibility or the transition of hits to a therapeutic strategy. The broken line represents the functional relationship in the absence of a linkage via a specific signaling network. The elucidation of the many metabolic processes that *KRAS* regulates may result in new therapies for patients with PDAC. Likewise, the discovery of ways to degrade the dense stroma associated with PDAC tumors and employ the immune response may lead to novel therapies for PDAC.

*KRAS* plays a vital role in PDAC and is believed to be a key target for treatment. Decades of research have shaped our understanding of the biochemistry, structure, and cellular signaling of KRAS in cancer. This foundation of knowledge can be viewed in two ways: support for the need to find different routes to silence *KRAS*, or fodder for the notion that *KRAS* is “undruggable”. In this review, the most promising paths taken in an attempt to suppress the effects of *KRAS* in cancer are discussed. We will examine efforts to target KRAS directly, prevent KRAS from binding to cellular membranes, inhibit its downstream effectors, search for synthetic lethal interactors of mutant *KRAS*, disrupt the metabolic pathways KRAS regulates, and exploit the ways KRAS signaling influences the tumor microenvironment (Figure 3).

## 2. Direct Inhibition of RAS

Inhibiting RAS directly is the most obvious approach and arguably the most attractive for developing a treatment for *KRAS* mutant PDAC. Since a treatment aimed at targeting all RAS isoforms would be deleterious to normal as well as neoplastic tissue, studies have focused on specifically targeting *KRAS*. Unfortunately, RAS activation and signaling is accomplished primarily through protein-protein interactions and such interfaces have traditionally been difficult to target with small molecules because they lack well-defined binding pockets [27]. Although some deemed KRAS “undruggable”, recent data have revived the hope to target RAS directly.

The first small molecules identified as direct binders that altered RAS function targeted the site on RAS involved in its recognition by the RASGEF, SOS1. SOS1 catalyzes the exchange of GDP to GTP, the rate-limiting step in RAS activation, and thus regulates RAS activity. Fragment-based lead discovery (FBLD) provided a starting point to finding compounds that bind to RAS targets, leading to the discovery of small molecules that bound to KRAS GDP and inhibited SOS-mediated nucleotide exchange [28]. Independently, a second group identified small molecules that bind to RAS and also inhibit SOS1-catalyzed nucleotide exchange [27]. These small molecules bind to the hydrophobic pocket on the CDC25 domain of SOS. The structure not only demonstrated how these small molecules bind, but also revealed other potential binding sites that have yet to be targeted by existing compounds [29].

Shortly after, another study identified molecules that inhibited RAS protein-protein interactions, the Kobe0065-family compounds were found to bind to RAS-GTP and inhibit interactions with downstream effectors [30]. Shima *et al.* suggests that once these compounds are structurally optimized, they could be used to develop RAS inhibitors with the high potency and specificity, as well as the low toxicity necessary for clinical application [30].

A third class of RAS-binding small molecules was developed to selectively recognize the G12C missense mutant of KRAS [31]. Targeting the S-IIP binding site affects KRAS signaling through two mechanisms. It shifts the nucleotide affinity from GTP to GDP, which leads to more RAS molecules in the inactive state, and it diminishes interactions with effectors [31]. SML-8-73-1, a GDP analogue has been developed to specifically target cancers with a KRAS^G12C^ mutation. SML-8-73-1 competes with GTP and GDP for active site binding and treatment with SML-8-73-1 stabilizes an inactive form of KRAS [32]. Although SML-8-73-1 can penetrate the cell membrane, it requires high concentrations, which may result in a loss of selectivity and potential off-target activities. Although the KRAS^G12C^ mutation is prevalent in non-small-cell lung cancer, this mutation is rarely found in PDAC (3%) [14]. To date, mutation specific compounds targeting the more common KRAS G12D or G12V mutations have yet to be developed.

Using RNA interference (RNAi) to suppress *KRAS* expression has been validated as therapeutic strategy in *RAS* mutant-driven mouse models of cancer [33,34]. RNAi mediated suppression of expression of mutant *KRAS* in pancreatic cancer cells reduced proliferation, anchorage-independent growth, and tumorigenic growth [10,35]. The effects of *KRAS* siRNA on PDAC suggest that RNAi can be explored as a potential drug for *KRAS* mutant PDAC [36]; however, the delivery of siRNA *in vivo* is a challenge because of enzymatic breakdown, renal clearance, and precise targeting to the tissue of interest. Improved delivery methods have been developed, such as a Local Drug EluteR, (LODER), a miniature biodegradable polymetric matrix that protects the siRNA and enables stable, local release of the siRNA for months within the tumor tissue [37]. Using this technology, delivery of *KRAS*^G12D^-specific siRNA clearly dampened *KRAS* expression and inhibited the *in vivo* growth of pancreatic tumors in both subcutaneous and orthotopic mouse models [37]. A clinical phase I/IIa study of siG12D-LODER in combination with chemotherapy was recently completed in patients with locally advanced PDAC [38]. The LODER was inserted into the tumor using a standard endoscope ultrasound biopsy and was thus able to provide local, continuous treatment in the tumor for several months. The treatment was given in combination with FOLFIRINOX, a standard of care chemotherapy cocktail commonly used in advanced pancreatic cancer patients in good health, and was tolerated well [38]. The results of the combination treatment showed a median overall survival of 15.13 months, and a median time to metastasis of 8.25 months [38]. A drawback to this technology is the LODER must be directly implanted in the tumor. Pecot *et al.* achieved systemic *in vivo* delivery of *KRAS*-targeting siRNA using a nanoliposomal delivery platform made of 1,2-dioleoyl-sn-glycero-3-phosphatidylcholine (DOPC). Mice with KRAS mutant lung cancer treated with the DOPC-mediated siRNA showed decreased downstream signaling, inhibited proliferation, and a decrease in metastatic burden *in vivo* [39]. Finally, since wild type and mutant *RAS* differ by just one missense mutation, an additional challenge is the development of siRNA that targets the mutant gene selectively.

## 3. Disruption of RAS Plasma Membrane Localization

RAS must be positioned at the inner face of the cell membrane to be biologically active, and the CAAX motif, located on all RAS isoforms, is both necessary and sufficient for a series of post-translational modifications that facilitate its membrane association [16,40]. Therefore, an attractive target for anti-RAS inhibitors is the prevention of post-translational lipid modification and membrane binding. The CAAX box of synthesized RAS proteins undergoes a series of modifications, the first step being the farnesyltransferase (FTase)-mediated covalent addition of a 15-carbon (C15) farnesyl isoprenoid to the cysteine of the CAAX-motif. Next, RAS-converting enzyme 1 (RCE1) catalyzes the proteolytic removal of the AAX peptide, and finally isoprenylcysteine methyltransferase (ICMT) catalyzes the carboxylmethylation of the now terminal farnesylated cysteine. Pharmacological inhibitors of all three CAAX-modifying enzymes have been developed, but FTase has been the most favored target since it is the first step of the three modifications [14]. Preclinical cell culture and mouse model studies showed farnesyl transferase inhibitors (FTIs) are potent, non-toxic inhibitors of HRAS-driven growth *in vitro* and *in vivo*. FTIs can also block the farnesylation and membrane association of the RAS isoforms more commonly mutated in cancer (KRAS and NRAS); however, when FTase activity is blocked, these RAS isoforms can undergo modification by a related lipid geranylgeranyl transferase enzyme (GGTase-I), overcoming the actions of FTI treatment. A logical solution to this limitation of FTIs is the use of GGTIs in combination treatment with FTI. However, since there may be up to 300 additional substrates of FTase and GGTase-I aside from RAS proteins, off-target effects are a concern for normal tissue toxicity *in vivo*.

Clinical trials of FTIs to treat PDAC and other cancers with prevalent *KRAS* mutations did not show significant anti-tumor activity or impact on patient survival [41,42]. A phase II study of tipifarnib in patients with surgically incurable or advanced PDAC showed no benefit [43]. The problem was initially thought to be the dose of tipifarnib, but further studies demonstrated that the dose was appropriate as inhibition of farnesyl transferase in peripheral blood monocytes was observed [44]. Since higher doses could not be tolerated, the possibility of combination therapies was explored, but a Phase III study with the combination of tipifarnib and the cytotoxic chemotherapy gemcitabine showed no benefits [45].

Another strategy to prevent the interaction of the RAS farnesyl group with the cell membrane is the use of a farnesyl-cysteine mimetic that would compete with RAS proteins for association with RAS anchorage cites [46]. Farnesyl thiosalicylic acid (FTS/salirasib) inhibits RAS signaling, in part, by dislodging RAS from the cell membrane and rendering it susceptible to proteolytic degradation [47]. Salirasib showed some promise in mice, as salirasib treatment inhibited cell growth in HRAS transformed rat fibroblasts with drug concentrations that did not affect processes like farnesylation and carboxyl methylation [48]. While salirasib showed activity towards active RAS, it is certain that salirasib will have non-RAS targets. Salirasib has undergone phase II clinical trials in non-small cell lung carcinoma (NSCLC) patients with mutated *KRAS*. The monotherapy was determined to be insufficient at the current dose with modest toxicity, presenting another disappointing result [49].

The discovery of proteins that facilitate the trafficking of RAS to the plasma membrane has provided an alternative approach to interfering with RAS membrane association. Phosphodiesterase 6 delta (PDEδ) is important for photoreceptor signaling and is responsible for the trafficking of the PDE6 complex (which contains farnesylated and geranylgeranylated substrates) [50]. PDEδ can also recognize KRAS4B and enhance its transit to the plasma membrane, and thus, interfering with the binding of PDEδ to KRAS provides an opportunity to disrupt RAS function [51]. Inhibition of the PDEδ-KRAS interaction using small molecules provides an opportunity to suppress KRAS and effect pancreatic cancer tumor development. Deltarasin, which is a high affinity PDEδ-KRAS interaction inhibitor, had a negative effect on the plasma membrane association of KRAS4B and reduced the growth of *KRAS*-dependent PDAC cell lines [51].

RAS controls many downstream pathways and this could be due to its compartmentalization in cells. In addition to the plasma membrane, RAS also signals from endosomes, the endoplasmic reticulum, the Golgi apparatus, and mitochondria. In T lymphocytes, the location of RAS signaling dictates the biological outcome [52]. Phosphorylation by protein kinase C (PKC) at serine 181 (S181) in the polybasic region of KRAS4B results in its release from the plasma membrane and accumulation of KRAS4B on internal membranes, representing a farnesyl-electrostatic switch [53]. After the switch is engaged, phospho-KRAS4B translocates from the cell membrane to the endoplasmic reticulum, Golgi apparatus and outer mitochondrial membrane [53]. This translocation is associated with antiproliferative effects as phospho-KRAS4B signaling through inositol-triphosphate receptors at the ER promotes cell death, suggesting a possible strategy for anti-RAS treatments [54].

Bryostatins are PKC agonists, and therefore are capable of triggering KRAS dissociation from the plasma membrane [55]. Mice bearing orthotopic tumors derived from the human pancreatic cancer cell line, KC1-MOH1 [56], responded very well to a combination of bryostatin-1 and gemcitabine with remission in only one of every seven animals [57]. However, to date, there have been more than 20 clinical studies using bryostatin-1 monotherapy or combination therapies in numerous different cancer types, and none have been successful [58]. In addition to not being clinically effective, there were toxic effects like myalgia, local phlebitis, fatigue, nausea, and thrombocytopenia [59]. Further studies determining the functionality of RAS based on its subcellular localization would be a critical step toward finding a drug that targets specific RAS pathways.

## 4. Searching for Synthetic Lethal Interactors

Synthetic lethality arises when a combination of mutations in two or more genes leads to cell death. Thus, synthetic lethal interactors of mutant RAS would be genes for which the loss of function would be lethal to the cell only in the presence of mutant RAS. The existence of oncogene-specific synthetic lethal interactions is supported by the notion that oncogenic transformation profoundly changes the phenotype of the cell [60,61]. Potential targets may exist is pathways that aid the *RAS*-transformed cell in coping with the cellular stress associated with persistent proliferation or the nutrient-supply pathways that fuel this proliferation. Several studies have identified synthetic lethal interactors with mutant *KRAS* through the use of RNAi screens in human cancer cell lines [62]; unfortunately, this first generation of screens yielded only new information about the biology of mutant *KRAS*-harboring cells, not new therapeutic targets.

The hits from screens for synthetic lethal interactors for mutant *KRAS* span many different cellular processes including: Cell cycle/mitosis, cell survival, gene transcription, and cell growth. Therapeutically, targeting cell cycle regulators such as survivin, *CDK1* [63], or *TPX2* [64] would most likely be similarly toxic to normal and neoplastic cells. Additionally, transcription factors such as *GATA2* [65,66] are largely considered undruggable. A potentially druggable hit, serine/threonine protein kinase 33 (STK33), was initially considered a tractable target [67]; however, follow-up studies have determined that both genetic depletion and pharmacological inhibition of STK33 has no effect on cell growth [68]. Likewise, genetic and pharmacological validation of the hit TBK1 [69] found no reproducible requirement for TBK1 in the growth of *KRAS*-mutant tumor cell lines *in vitro* [70].

There were a number of limitations with the first generation of mutant *KRAS* synthetic lethal screens that could be improved upon in future studies. First, many of these screens relied on isogenic matched pairs of cells lines harboring *KRAS* mutations, and a matched counterpart in which the *KRAS* allele is genetically ablated. Acute ablation of *KRAS* causes apoptosis and severe growth impairment [71], and thus proliferating cells that eventually arise must acquire additional, adaptive alterations and are therefore not truly isogenic. Currently, efforts are underway to screen large panels of cancer cell lines that are more representative of the heterogeneity that exists in human *KRAS*-mutant cancers. Additionally, all previously described screens have utilized *in vitro* anchorage-dependent culture conditions. Second generation screens would benefit from anchorage-independent culture systems, such as organoid cultures or *in vivo* xenograft tumor assays, which more accurately model tumor biology. Such methods have already been utilized in screens for other purposes [72,73,74]. Furthermore, the first generation screens serve as a reminder that as with any high-throughput approach, hits must be rigorously validated.

## 5. Targeting RAS Downstream Signaling Pathways

Eleven RAS effector families have been identified to date, with six of these families having validated roles in contributing to RAS-dependent cancer initiation and/or maintenance [17,75]. As directly targeting the RAS protein has proved challenging, currently the most favorable method for targeting RAS signaling is through targeting its downstream effectors’ signaling. Herein, we will focus on the most intensely targeted pathways: the RAF-MEK-ERK mitogen-activated protein kinase (MAPK) cascade and the PI3K-AKT-mTOR cell-survival signaling pathway.

### 5.1. RAF-MEK-ERK

Active RAS can engage three highly related RAF serine/threonine kinases: ARAF, BRAF, and CRAF. The largely mutually exclusive frequency of *BRAF* and *RAS* mutations supports the notion that RAF is a critical driver in *KRAS*-mutant pancreatic cancer. RAS-GTP binds preferentially to RAF, resulting in the translocation of RAF to the plasma membrane where subsequent events promote its activation. Active RAF phosphorylates and activates the MEK1 and MEK2 dual specificity kinases, which in turn phosphorylate and activate ERK1 and ERK2 serine-threonine MAPKs. Activated ERKs phosphorylate a diverse spectrum of more than 200 cytoplasmic and nuclear substrates [76,77].

There are currently two BRAF-selective inhibitors in the clinic, vemurafenib and dabrafenib. Vemurafenib is approved for the treatment of *BRAF*-mutant melanoma. Vemurafenib proved effective in melanoma patients harboring *BRAF* mutations with a response rate of greater than 50% and a rapid improvement in quality of life. Unexpectedly, when these RAF inhibitors were used in *RAS*-mutant cancers, activation rather than inactivation of ERK was observed [78,79,80]. This is due to the propensity of these first-generation RAF inhibitors to induce RAF dimerization, which causes activation of CRAF [81]. Recently, pan-RAF inhibitors have entered clinical evaluation [82]. This new class of inhibitors is not subject to the paradoxical activation seen with the BRAF-selective inhibitors, and may be more effective in *RAS*-mutant cancers [83,84].

In addition to BRAF inhibitors for melanoma, MEK inhibitors have also be developed. Trametinib (GSK112021) is a selective allosteric inhibitor of MEK1/2 activation and kinase activity [85]. In a Phase III trial of patients with advanced or metastatic *BRAF^V600E/K^*-positive melanoma, the response rate for the trametinib monotherapy was 22%, but in combination with dabrafenib the response rate increased to 64% [85]. This combination therapy likely delays pathway alterations that lead to ERK reactivation and the resistance that occurs in response to BRAF or MEK monotherapy. While MEK inhibitors have shown success in the treatment of *BRAF*-mutant melanoma, they have shown limited efficacy in *RAS*-mutant human tumor cell lines [86] and *RAS*-driven mouse models of cancer [87]. A recent study suggests that the mechanism by which MEK is activated in *RAS*-versus *BRAF*-mutant cancers is different, thus explaining the different responses in different systems [88]. Resistance to MEK inhibitors in *RAS*-driven cancers occurs due to upregulation or amplification of upstream activators that restore ERK activity [82].

As dynamic kinome reprogramming [89] in response to RAF and MEK inhibitors eventually leads to increased ERK signaling in *RAS*-driven cancers, ERK has become an attractive target [81,82]. Four ERK inhibitors (BVD-523, MK8353, GDC-0994, and CC-90003) have entered Phase I studies, and MK8353 (an analog of SCH772984) has been described preclinically [90], where it showed promising results in BRAF- or MEK-inhibitor resistant cell line models [90]. Additionally, concurrent targeting of the RAF-MEK-ERK cascade at multiple nodes is currently under investigation as an effective strategy to achieve prolonged ERK suppression. A recent study focused on directly targeting ERK as a treatment for pancreatic cancer identified the degradation of the MYC oncoprotein and the induction of a senescence-like phenotype as the predominant growth suppression mechanism of ERK inhibitors [71]. This study also identified PI3K-AKT-mTOR signaling as a critical determinant of ERK inhibitor sensitivity, and PI3K, Notch, and p38 as potential modulators of ERK resistance [71]. This suggests that multiple inhibitor-based combinations will be necessary to treat across multiple *KRAS*-mutant PDAC populations.

### 5.2. PI3K-AKT-mTOR

The catalytic subunits of class I PI3K lipid kinases (α, β, δ and γ) comprise the second-best validated effector family critical for *RAS*-driven cancer growth. The gene encoding PI3Kα (*PIK3CA*) is often mutationally activated in cancers, which supports its role as a cancer driver. PI3K signaling pathways are important for the regulation of cellular functions such as metabolism, growth, proliferation, survival, transcription and protein synthesis. Mutant RAS activates PI3K. Once PI3K is activated, it binds to PIP_2_ (phosphatidylinositol 4,5-bisphosphate), a component of the cell membrane and phosphorylates PIP_2_ to PIP_3_ (phosphatidylinositol 3,4,5-triphosphate), which in turn can regulate the activities of many signaling proteins, in particular the 3-phosphoinositide-dependent protein kinase-1 (PDPK1 or PDK1) and AKT serine/threonine kinase. Upon PIP_3_-dependent recruitment to the plasma membrane, AKT is phosphorylated by PDK1, which itself is associated with PIP_3_ at the plasma membrane. AKT promotes activation of the Rheb small GTPase, which then activates mTOR, a protein that is involved in growth factor signaling, the energy state of the cell, and nutrient and oxygen availability.

There are four main pharmacologic approaches for inhibition of PI3K signaling: PI3K inhibitors, AKT inhibitors, and mTOR inhibitors, and dual PI3K-mTOR inhibitors [14,91]. PI3K inhibitors can be isoform-specific or pan-PI3K inhibitors, which target all class I PI3Ks. Whether pan- or isoform-specific PI3K inhibitors will be more effective is not clear. Isoform-selective treatment may exhibit less toxicity, which means it may be tolerated at the higher doses necessary for more complete target inhibition with fewer adverse effects [92]. The realization that different PI3K isoforms play non-redundant roles in different tumor types has attracted increasing interest in isoform-specific inhibitors [93]. However, since RAS can utilize multiple PI3K isoforms, more effective suppression may require a pan-PI3K inhibitor. More work must be done to understand the mechanisms underlying drug resistance and escape of PI3K dependency following isoform-specific therapies. AKT inhibitors are typically either ATP mimetics or allosteric, non-catalytic site inhibitors. Allosteric AKT inhibitors block the attachment of AKT to the membrane by interfering with the binding of the PH (pleckstrin homology) domain to phosphoinositides. Mislocalization of AKT in turn diminishes its ability to signal. A potential drawback of this class of inhibitors is that they will not block the non-AKT effectors of PI3K signaling and hence paradoxically increase the PI3K-dependent activation of those effectors via the loss of negative feedback [92].

Mammalian target of rapamycin (mTOR) exists as two distinct complexes, mTOR complex 1 (mTORC1; which contains the regulatory-associated protein of TOR1 (RAPTOR)) and mTORC2 (which contains the rapamycin-insensitive companion of mTOR (RICTOR)). Rapamycin and its analogues (also known as rapalogues, which include everolimus, ridaforolimus and temsirolimus) are selective for mTORC1, forming an inactive complex with mTOR and FKBP12. Second-generation mTOR catalytic site inhibitors directly inhibit both mTOR complexes, mTORC1 and mTORC2, and are more effective inhibitors of downstream signaling and ultimately RNA translation than the first generation rapalogues [94,95]. A concern with these inhibitors is that feedback activation of PI3K from mTOR inhibition may result in hyperactivation of AKT-independent effectors of PI3K signaling [92]. Since the p110 subunits of PI3K and mTOR have similar structures, the inhibition of p110 often results in the inhibition of mTOR [96]. This dual inhibition of PI3K-mTOR is expected to shut down PI3K-AKT-mTORC1 signaling; however, it is still unclear whether a dose that sufficiently blocks cellular signaling will be tolerable.

The recent observation that downstream of PI3K, PDK1 is a key effector of oncogenic KRAS signaling in the pancreas has led to enhanced interest in specifically targeting this PI3K effector [97]. PHT-427 is a novel AKT/PDK1 pleckstrin homology domain inhibitor, which is capable of binding to both AKT and PDK1; however, inhibition of PDK1 was more closely correlated to its antitumor activity than AKT inhibition [98]. Furthermore, when PHT-427 was encapsulated in poly (lactin-co-glycolic) acid (PLGA) nanoparticles drug delivery was improved and tumor volume was reduced by 4-6 fold in preclinical mouse models [99].

Monotherapies targeting PI3K, AKT, and/or mTOR have been largely disappointing in *RAS*-mutant cancers. However, in mouse models, potent synergistic activity has been observed when inhibitors of the PI3K pathway are combined with inhibitors of the RAF-MEK-ERK cascade [87]. Specifically, in mice with PDAC, treatment with MEK (GDC-0973) or PI3K inhibitors (GDC-0941) alone showed slight tumor growth inhibition and had no significant effect on survival. However, in comparison to the monotherapies, the combination of the two treatments resulted in a survival advantage [100]. Furthermore, combined inhibition of MEK and AKT showed synergistic activity in PDAC cell lines *in vitro* [101]. There are now numerous clinical trials evaluating the effect of combined inhibition of PI3K and RAF [102]. Recently, a novel approach for targeting these two pathways was described. Van Dort *et al.* designed a single compound that is a hybrid of the ATP competitive pan-PI3K inhibitor ZSTK474 [103] and the allosteric MEK inhibitor RO5126766 [104]. Western blot analysis showed a dose-dependent decrease of pERK and pAKT in treated PANC-1 cells, verifying the compound is cell permeable and effective [104]. Although this therapy must be optimized in order to achieve MEK and PI3K inhibition in mouse models, it is an important framework for creating a combination therapy with a single compound [104].

## 6. *KRAS*-Regulated Metabolic Targets

Oncogenic *KRAS* has been implicated in controlling a number of metabolic processes including induction of glucose uptake, unique utilization of glucose intermediates, repurposed glutamine metabolism, and increased autophagy and macropinocytosis [13]. Further elucidation of the links between oncogenic *KRAS* and deregulated PDAC metabolism has the potential to result in the formulation of new anti-KRAS therapies.

### 6.1. Glucose Utilization and Glutamine Metabolism

PDAC cells have altered metabolic processes consistent with increased aerobic glycolysis [105]. Interestingly, it has been demonstrated that in PDAC, mutant *KRAS* is responsible for orchestrating this phenotype by enhancing the expression of the glucose transporter *GLUT1*, as well as many other genes that encode rate-limiting glycolytic enzymes, including hexokinase I and 2 (HK1, HK2), phosphofrutokinase-1 (PFK1), and lactate dehydrogenase A (LDHA) [8]. This regulation could be exploited therapeutically by targeting LDHA, as was demonstrated using a small molecule (FX-11), which caused increased ROS production and cell death [106]. Hk2 has been identified as an attractive target for *KRAS*-driven lung cancers as whole-body deletion of *Hk2* in the mouse selectively targets tumor cells [107]. The small molecule 3-bromopyruvate (3BP) inhibits Hk2 and has shown potent anticancer activity in a number of animal models [108], as well as promising results in a human case study [109]. Additionally, mutant *KRAS* expression leads to the shunting of glycolytic intermediates through the non-oxidative arm of the pentose phosphate pathway (PPP), which leads to the generation of ribose 5-phosphate, a necessary component for nucleic acid biosynthesis [8]. Downstream of mutant KRAS, the ERK-MAPK pathway, which culminates with the MYC transcription factor, is the major driver of glucose metabolism adjustments [8].

In addition to altered levels of glycolysis, cancer cells also display an increased dependence on glutamine [110], which contributes to cancer cell proliferation by providing carbon to fuel the tricarboxylic acid (TCA) cycle and nitrogen for nucleotide, nonessential amino acid, and hexosamine biosynthesis [111]. Glutamine is catabolized to α-ketoglutarate (αKG), a TCA cycle intermediate, through two deamination reactions, the first requiring glutaminase (GLS) to generate glutamate and the second occurring via glutamate dehydrogenase (GDH) or transaminases. mTORC1 has been shown to positively regulate GLS and glutamine flux through this pathway through the S6K1-dependent regulation of MYC [112]. *KRAS* mutant PDAC has been shown to utilize glutamine metabolism to regulate redox balance by increasing the NADPH/NADP^+^ ratio in the cell through an aspartate transaminase (GOT1)-dependent mechanism [113]. As treatment with glutamine analogs is profoundly toxic [114] the current strategy for targeting glutamine utilization as a cancer treatment, is to target those processes that cancer cells are specifically addicted to. Thus, as GOT1 is dispensable for normal cells while PDAC cells rely on this enzyme for redox homeostasis, it could be an enticing therapeutic target [113]. Additionally, two previously described GLS inhibitors Compound 968 [115] and bis-2-(5-phenylacetamido-1,2,4-thiadiazol-2-yl)ethyl sulfide (BPTES) [116], have demonstrated a growth suppressive effect on PDAC cells that is enhanced when combined with hydrogen peroxide treatment [113]. Finally, as inhibition of GOT1 or GLS ultimately leads to a disruption of redox homeostasis in PDAC, such inhibition may synergize with therapies that increase reactive oxygen species, such as chemotherapy and radiation [117].

### 6.2. Macropinocytosis and Autophagy

To fuel metabolic processes, *KRAS* signaling leads to the scavenging of extracellular proteins and lipids and activates self-eating and recycling of proteins through autophagy [118]. PDAC cells specifically expressing oncogenic *KRAS* utilize macropinocytosis to transport extracellular protein into the cell [119] and use it as a source of essential amino acids (EAAs) in order to sustain survival and proliferation [120]. Consistent with these studies, active macropinocytosis has been observed in primary human PDAC specimens [121]. This scavenging phenotype appears to be a general property of *RAS*-driven cancers as *RAS*-transformed cells have also been shown to scavenge lysophospholipids, which contributes to their metabolic robustness [121]. The regulation of the degradation of the EAAs taken up by macropinocytosis may shed light on the disappointing lack of efficacy of mTOR inhibitors as PDAC therapeutics. It has been demonstrated that in mammalian cells, mTORC1 signaling suppresses lysosomal catabolism of proteins that were taken up from the extracellular environment [120]. Thus, mTORC1 inhibition may enhance cell proliferation that depends on extracellular proteins, such as PDAC cells inhabiting a poorly vascularized area [120]. A specific inhibitor of macropinocytosis has yet to reach the clinic; however, preclinical studies in which heterotopic tumor bearing mice were treated with the tool compound 5-(*N*-ethyl-*N*-isopropyl) amiloride (EIPA) showed attenuation of tumor growth and in some cases, regression [119].

Autophagy is a highly conserved mechanism to degrade intracellular components and promote the survival of stressed cells by providing energy in the form of ATP and building blocks such as amino acids, lipids, sugars, and nucleosides [122]. The role of autophagy in cancer is extremely complex [123] and while it appears clear that PDAC cells depend on autophagy for growth, the role of oncogenic *KRAS* in this dependence remains unclear. When tissue samples from 71 PDAC patients were analyzed via immunohistochemical staining for LC3 protein (a component of the autophagosome), it was determined that high expression was correlated with large tumor size, short-disease free period, and overall poor patient outcome [124]. Additional studies have revealed that pancreatic cancers have a clear dependence on autophagy. Genetic or pharmacological inhibition of autophagy results in increased reactive oxygen species, elevated DNA damage, and mitochondrial defects that lead to decreased proliferation of pancreatic cancer cell lines *in vitro*, as well as substantial tumor regression and sustained survival in *in vivo* models of pancreatic cancer [125,126]. In support of a cooperative role between *RAS* expression and proliferation fueled by autophagy, immortal, non-tumorigenic baby mouse kidney epithelial (iBMK) cells ectopically expressing oncogenic *HRAS* or *KRAS* experienced defects in mitochondrial respiration upon autophagy inhibition [126]. However, a cooperative relationship between RAS and autophagy is not universally reported. For example, acute expression of oncogenic HRAS^G12V^ in immortalized human ovarian surface epithelial cells was associated with caspase-independent cell death, rather than increased proliferative capacity [127]. Furthermore, autophagy was implicated as a facilitator of *RAS*-induced senescence in a study in which oncogenic HRAS was overexpressed in IMR90 human diploid fibroblasts [128]. Additionally, a very recent study assayed a panel of 47 different cancer cell lines comprised of both *KRAS* mutant and *KRAS* wild-type lines and found that the *KRAS*-mutated cells were no more dependent on autophagy than their wild-type counterparts [129]. Thus, the specific role that *KRAS* plays in the upregulation of autophagy in PDAC remains a controversy.

A recent study that used a mouse model of PDAC harboring an embryonic homozygous *Trp53* deletion paradoxically demonstrated that loss of autophagy accelerates tumor onset [130]. However, further studies using a mouse model with *Trp53* loss of heterozygosity, which is similar to *TP53* mutations in human PDAC, as well as patient-derived xenographs showed that p53 status does not affect the response of a patient to autophagy inhibition [131]. Interestingly, an inhibitor of autophagy, hydroxychloroquine (HCQ), has been available clinically for quite some time as the FDA approved it for the treatment of malaria and rheumatic disorders years ago. Hence, there are multiple early phase studies exploring the use of HCQ in as a treatment for pancreatic cancer. However, early observations have been disappointing, likely due to the limited potency of HCQ to block autophagy *in vivo* [132]. Notably, HCQ does not specifically inhibit autophagy, it is an inhibitor of lysosomal acidification, and thus hinders all pathways that terminate in the lysosome. Therefore, processes such as macropinocytosis are similarly inhibited with HCQ. This is supported by a recent study that found that while HCQ treatment is antiproliferative and synergizes with targeted anticancer drugs, these effects may be independent of autophagy inhibition [129].

## 7. Harnessing the Immune Response

Recent successes of cancer immunotherapy, such as antibody blockade of cytotoxic T lymphocyte antigen-4 (CTLA-4), have generated a lot of excitement, but unfortunately this form of treatment has been less successful in patients with pancreatic cancer [133]. A major barrier for immunotherapeutic approaches is thought to be profound immune suppression associated with the pancreatic tumor microenvironment [134]. It is now well understood that formation of PDAC is accompanied by pronounced alterations in stromal responses and immune surveillance programs, and there is an increasing appreciation that signaling by oncogenic RAS plays a direct role in orchestrating some of these changes in tumor microenvironment. Multiple signaling mechanisms downstream of RAS may account for this effect. Activation of oncogenic RAS has been shown to down regulate expression of major histocompatibility complexes (MHC) [135,136,137,138], resulting in decreased antigen presentation by tumor cells and reduced recognition by the immune system [139,140]. Oncogenic RAS has also been shown to upregulate expression of immunomodulatory cytokines, such as IL-8 and GM-CSF. Experimental perturbation in tumor-derived cytokine levels resulted in reduced inflammation and increased anti-tumor cytotoxic T cell response [141,142]. Significantly, pharmacologic inhibition of either ERK or AKT downregulated cytokine expression in KRAS transformed cells. These observations propel the hypothesis that abrogation of signaling pathways downstream of activated RAS may improve anti-tumor immune response. In support of this idea, inhibition of MEK or BRAF in melanoma correlated with reduced levels of immunosuppressive cytokines and an increase in infiltrating T cells [143,144,145]. A rational extension of this hypothesis is the idea that inhibition of RAS signaling may yield better responses to immune checkpoint blockade agents. Indeed, recent studies in TBNC featuring activation of RAS signaling pathways demonstrated that co-targeting of MEK and PD-L1 results in upregulation of MHC I and II on tumor cells and increase in CD8 T cell infiltration [146]. While inhibition of RAS-driven signaling has so far been shown to have positive immunomodulatory function, it will also be helpful to evaluate its potential effects on T cell activity [147]. Overall, strategies aimed at combining RAS-targeted therapy and immunotherapy hold significant promise as clinically feasible and effective therapeutic modalities.

PDAC is distinctive as desmoplastic stroma accounts for 70%–80% of the tumor volume [148,149]. Stromal accumulation is *KRAS* driven and is initiated in PanIN lesions, suggesting that its early onset is important to tumor growth and progression [150]. The dense stromal compartment is thought to prevent the delivery of drugs to the target tissue; therefore, the targeting of the stroma itself may be a way to improve current chemotherapeutics. The stroma is composed of cancer-associated fibroblasts, extracellular matrix, inflammatory cells and blood vessels [151]. Stroma and cancer cells both contribute to the extracellular matrix, which is composed of macromolecules like collagens and hyaluron and regulatory components like secreted protein acidic and rich in cytosine (SPARC) [152]. Hyaluronic acid is trapped in the interstitium of PDAC, where it can reach one of the highest concentrations found in nature [153]. Here, it absorbs water and significantly increases the interstitial fluid pressure. Targeting hyaluronic acid with hyaluronidase (PEGPH20) reduces HA, thereby reducing interstitial pressure and supporting drug delivery [154]. In combination with gemcitabine, PEGPH20 increased response rate, decreased metastasis and increased median survival in the KPC mouse model of PDAC [154]. Clinical trials have suggested the co-treatment provides improved progression-free survival for PDAC patients with high hyaluron expression, while increasing chances of thromboembolic events [155]. Although the pursuit of stromal depletion appears promising, other recent data suggest that the stroma could also function to protect the cancer from quickly spreading [152]. Clearly, PDAC has a unique microenvironment due to its unusual stromal content, which requires a better understanding.

## 8. Conclusions

In summary, we have highlighted six promising paths to finding an effective treatment for *KRAS*-mutant pancreatic cancer: targeting *KRAS* directly, upsetting its membrane association, exploiting synthetic lethal interactions, targeting the pathways downstream of *KRAS*, pursuing the metabolic processes that *KRAS* regulates, and harnessing the immune response. Although each of these approaches has shown promise in cells and even animal studies, none of these treatments have been very successful in the clinic. Thus, pancreatic cancer is projected to overcome breast cancer to become the third leading cause of cancer deaths in the U.S. in 2016 [156], and then surpass colorectal cancer to become the second leading cause of cancer deaths in the U.S. by around 2020 [157]. The major challenge with treatments that seem successful preclinically is that a patient quickly develops resistance, making combination therapies an attractive new direction. Despite the lack of clinical treatments to date, studies to better understand *RAS* mutant cancers continue to move in promising directions, which will hopefully lead to benefits for patients with PDAC.

## Abbreviations

The following abbreviations are used in this manuscript:

3BP	3-bromopyruvate
BPTES	bis-2-(5-phenylacetamido-1,2,4-thiadiazol-2-yl)ethyl sulfide
CDK	cyclin-dependent kinase
CDKN2A	cyclin-dependent kinase inhibitor 2A
CTLA-4	cytotoxic T lymphocyte antigen-4
DOPC	1,2-dioleoyl-sn-glycero-3-phosphatidylcholine
EAA	essential amino acid
EIPA	5-(N-ethyl-N-isopropyl)amiloride
ER	Endoplasmic Reticulum
FBLD	fragment-based lead discovery
FTase	farnesyltransferase
FTI	farnesyl transferase inhibitor
FTS	farnesyl thiosalicylic acid
GAP	GTPase activating proteins
GDP	guanosine diphosphate
GEF	guanine nucleotide exchange factors
GTP	guanosine triphosphate
HA	hyaluronic acid
HBS	hydrogen bond surrogate
HCQ	hydroxychloroquine
Hk1	hexokinase 1
Hk2	hexokinase 2
ICMT	isoprenylcysteine methyltransferase
LDHA	lactate dehydrogenase A
LODER	Local Drug EluteR
MHC	major histocompatibility complexes
mTOR	mammalian target of rapamycin
mTORC1	mTOR complex 1
mTORC2	mTOR complex 2
NSCLC	Non-small cell lung cancer
PanIN	pancreatic intraepithelial neoplasia
PDAC	Pancreatic Ductal Adenocarcinoma
PDEδ	Phosphodiesterase δ
Pfk1	phosphofructokinase
PFS	progression free survival
PH	pleckstrin homology
PIP_2_	phosphatidylinositol 4,5-bisphosphate
PKC	protein kinase C
PPP	pentose phosphate pathway
RCE1	RAS converting enzyme 1
RNAi	RNA interference
Serine 181	S181
SOS	Son of Sevenless
TP53	tumor protein 53
